# Altered DNA methylation pattern reveals epigenetic regulation of *Hox* genes in thoracic aortic dissection and serves as a biomarker in disease diagnosis

**DOI:** 10.1186/s13148-021-01110-9

**Published:** 2021-06-08

**Authors:** Peiru Liu, Jing Zhang, Duo Du, Dandan Zhang, Zelin Jin, Wenqing Qiu, Xiushi Zhou, Shulong Dong, Mengyu Zhou, Heyu Zhao, Wei Zhang, Jiakang Ma, Shaoyang Sun, Weiguo Fu, Yun Liu, Lixin Wang

**Affiliations:** 1 MOE Key Laboratory of Metabolism and Molecular Medicine, Department of Biochemistry and Molecular Biology, School of Basic Medical Sciences and Zhongshan Hospital, Fudan University, Shanghai, People’s Republic of China; 2 Vascular Surgery Department, Zhongshan Xiamen Hospital, Fudan University, Xiamen, People’s Republic of China; 3 Vascular Surgery Department, Zhongshan Hospital, Fudan University, Shanghai, People’s Republic of China; 4 Department of Cardiac Surgery, Shanghai Chest Hospital, Shanghai Jiao Tong University, Shanghai, People’s Republic of China; 5 State Key Laboratory of Medical Neurobiology and MOE Frontiers Center for Brain Science, Institutes of Brain Science, Fudan University, Shanghai, People’s Republic of China

**Keywords:** Aortic dissection, DNA methylation, Epigenetics, Homeobox genes, Cell-free DNA, Epigenetic biomarker

## Abstract

**Background:**

Thoracic aortic dissection (TAD) is a severe disease with limited understandings in its pathogenesis. Altered DNA methylation has been revealed to be involved in many diseases etiology. Few studies have examined the role of DNA methylation in the development of TAD. This study explored alterations of the DNA methylation landscape in TAD and examined the potential role of cell-free DNA (cfDNA) methylation as a biomarker in TAD diagnosis.

**Results:**

Ascending aortic tissues from TAD patients (Stanford type A; *n* = 6) and healthy controls (*n* = 6) were first examined via whole-genome bisulfite sequencing (WGBS). While no obvious global methylation shift was observed, numerous differentially methylated regions (DMRs) were identified, with associated genes enriched in the areas of vasculature and heart development. We further confirmed the methylation and expression changes in homeobox (Hox) clusters with 10 independent samples using bisulfite pyrosequencing and quantitative real-time PCR (qPCR). Among these, HOXA5, HOXB6 and HOXC6 were significantly down-regulated in TAD samples relative to controls. To evaluate cfDNA methylation pattern as a biomarker in TAD diagnosis, cfDNA from TAD patients (Stanford type A; *n* = 7) and healthy controls (*n* = 4) were examined by WGBS. A prediction model was built using DMRs identified previously from aortic tissues on methylation data from cfDNA. Both high sensitivity (86%) and specificity (75%) were achieved in patient classification (AUC = 0.96).

**Conclusions:**

These findings showed an altered epigenetic regulation in TAD patients. This altered epigenetic regulation and subsequent altered expression of genes associated with vasculature and heart development, such as Hox family genes, may contribute to the loss of aortic integrity and TAD pathogenesis. Additionally, the cfDNA methylation in TAD was highly disease specific, which can be used as a non-invasive biomarker for disease prediction.

**Supplementary Information:**

The online version contains supplementary material available at 10.1186/s13148-021-01110-9.

## Background

Thoracic aortic dissection (TAD) is a devastating disease with high mortality and severe complications. Based on aortic location, TAD can be clinically classified into Stanford type A (ascending aorta) or type B (descending aorta). Patients with Stanford type A generally require emergent surgical repair, with about half experiencing mortality if treatment is not obtained within three days of aortic tearing [1]. Although several genetic mutations have been found to be associated with some types of familial and syndromic TAD [2–4], mechanisms pertaining to the initiation and progression of TAD are poorly understood. Furthermore, little is known about the pathogenesis of sporadic TAD, which are the prevalent forms within the Chinese population. Recent evidence has suggested that life-style, environmental settings, and other risk factors (e.g., age and hypertension) may all contribute to TAD onset and course [5–7]. However, the precise mechanisms that modulate such intrinsic and extrinsic factors and the influence they have on TAD development is still largely unknown.

Since the high mortality rates of TAD, immediate early diagnoses are critical. Currently, TAD diagnoses mostly rely on imaging techniques, such as computed tomographic angiography (CTA), magnetic resonance imaging (MRI) or digital subtraction angiography (DSA), which may not be available in remote area or in certain emergent situation. Recent studies showed that D-dimer and sST2 can be used in blood test for TAD diagnoses, but still with low sensitivity [8, 9]. Methylation pattern in cell-free DNA has showed high specificity and sensitivity in some disease detections [10–12], and thus, it may be used as a potential biomarker for TAD diagnosis.

Epigenetics is the study of heritable changes that regulate gene expression without altering the DNA sequence itself. Numerous recent studies have shown that altered epigenetic regulations, such as DNA methylation, are associated with the development of tumors [13], metabolic diseases [14] and autoimmune disorders [15]. DNA methylation has also been shown to play a role in cardiovascular diseases (CVDs), such as heart failure [16], aberrant cardiovascular development [17] and mineralization of the aortic valve [18], where extrinsic risk factors affect gene expression by altering methylation and impacting downstream molecular events. However, few studies have examined epigenetic regulations in TAD, especially for DNA methylation.

In this study, we explored the genome-wide DNA methylation alteration in Stanford type A TAD ascending aortic tissues relative to healthy controls, identified numerous differentially methylated regions (DMRs) and investigated the gene expression changes. In addition, we constructed a prediction model using DMRs identified from aortic tissues on methylation signature of cell-free DNA (cfDNA) for TAD identification.

## Results

### Pathological alterations in dissected thoracic aortic tissues

Ascending aortic tissues from Stanford type A TAD patients and healthy controls were collected, with the adventitia removed to avoid any contributions from associated fatty tissues or endothelial cells. Histopathologic examination revealed similar structural components between the TAD and control samples. The dissected thoracic tissues from both groups were predominantly composed of smooth muscle cells (SMCs), collagens and elastic fibers (Additional file 1: Figures S1A and S1B). Despite both groups being predominantly comprised of SMCs, structural protein alterations associated with pathology were observed. TAD samples showed an elevated collagen deposition (Additional file 1: Figure S1C and S1D) and a severe fragmentation of the elastic fibers (Additional file 1: Figures S1E and S1F) when compared to the control samples.

### Genome-wide DNA methylation analysis of aortic tissues

To evaluate whether any epigenetic alterations are associated with TAD pathogenesis, ascending aortic tissues from six TAD (Stanford type A) and six healthy controls were examined using WGBS. Following quality control and data preprocessing, one control sample with bisulfite conversion rate less than 90% (sample: N2) was eliminated. Among the 11 remaining samples, no significant global DNA methylation alterations were noted between the TAD and control samples (Additional file 1: Table S2).

**Fig. 1:**
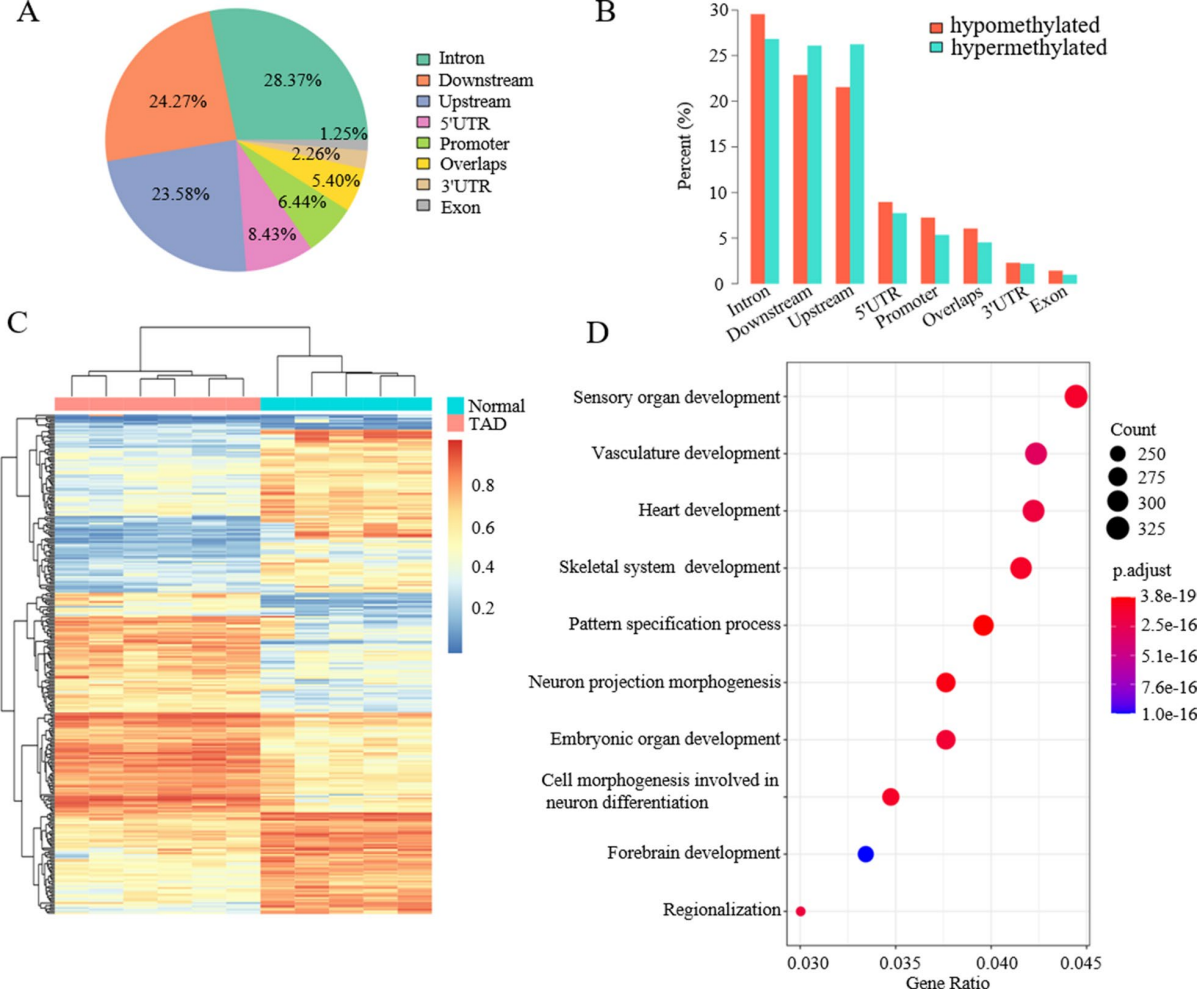
DNA methylation differences between TAD and healthy control samples. **a** DMR percentages in different genomic components (promoters were defined as 1–2,500 bp upstream of transcription start site, alternative promoters were not included.). **b** Hypermethylated and hypomethylated DMR percentages in different genomic components. **c** Supervised clustering with the top 300 DMRs (detail information was showed in Additional file 2: Table S3). **d** Gene ontology (GO) enrichment analysis with a focus on biological processes for the hypomethylated DMRs

Based on these findings, DMR analysis was performed to identify epigenetic differences between the two groups. A total of 51,468 DMRs (22,318 hypermethylated and 29,150 hypomethylated) were identified in the TAD samples relative to the controls, with 3314 of them (6.44%) located in promoter regions (1 to 2500 bp upstream of transcription start site) (Fig. 1a). Among them, the percentages of hyper- and hypomethylated DMRs were similar in all of the eight examined gene annotation groups (Fig. 1b). The top 300DMRs (Additional file 2: Table S3) included 14,365 CpGs with the median length of 1648 bp. Most of them were located at non-gene regions, the median distance to nearest gene was 58,305 bp. By using the top 300 DMRs (Additional file 2: Table S3), we were able to separate samples into two groups corresponding to TAD and the controls (Fig. 1c), thus suggesting that these DMRs may be potential biomarkers for TAD. To characterize the functional relevance of these DMRs, all DMRs were associated with their nearest gene and the gene ontology and pathway enrichment analysis was performed. The hypomethylated DMRs were found to be significantly enriched in the areas of vasculature development (adjusted *P* value = 3.13 × 10^−16^) and heart development (adjusted *P*-value = 1.81 × 10^–16^; Fig. 1d, results for hypermethylated DMRs are shown in Additional file 1: Figure S2). Moreover, MAPK signaling pathway and calcium signaling pathway were enriched in hypomethylated or hypermethylated DMRs based on Kyoto Encyclopedia of Genes and Genomes (KEGG) (Additional file 1: Figure S3). The abnormal activation of these two pathways involved in vascular remodeling was reported to contribute to aortic dissection or aneurysm formation [19, 20]. These findings suggest that TAD is associated with a possible epigenetic dysregulation in genes associated with vessel development and aortic integrity.

### Altered DNA methylation of *Hox* genes in TAD

**Fig. 2:**
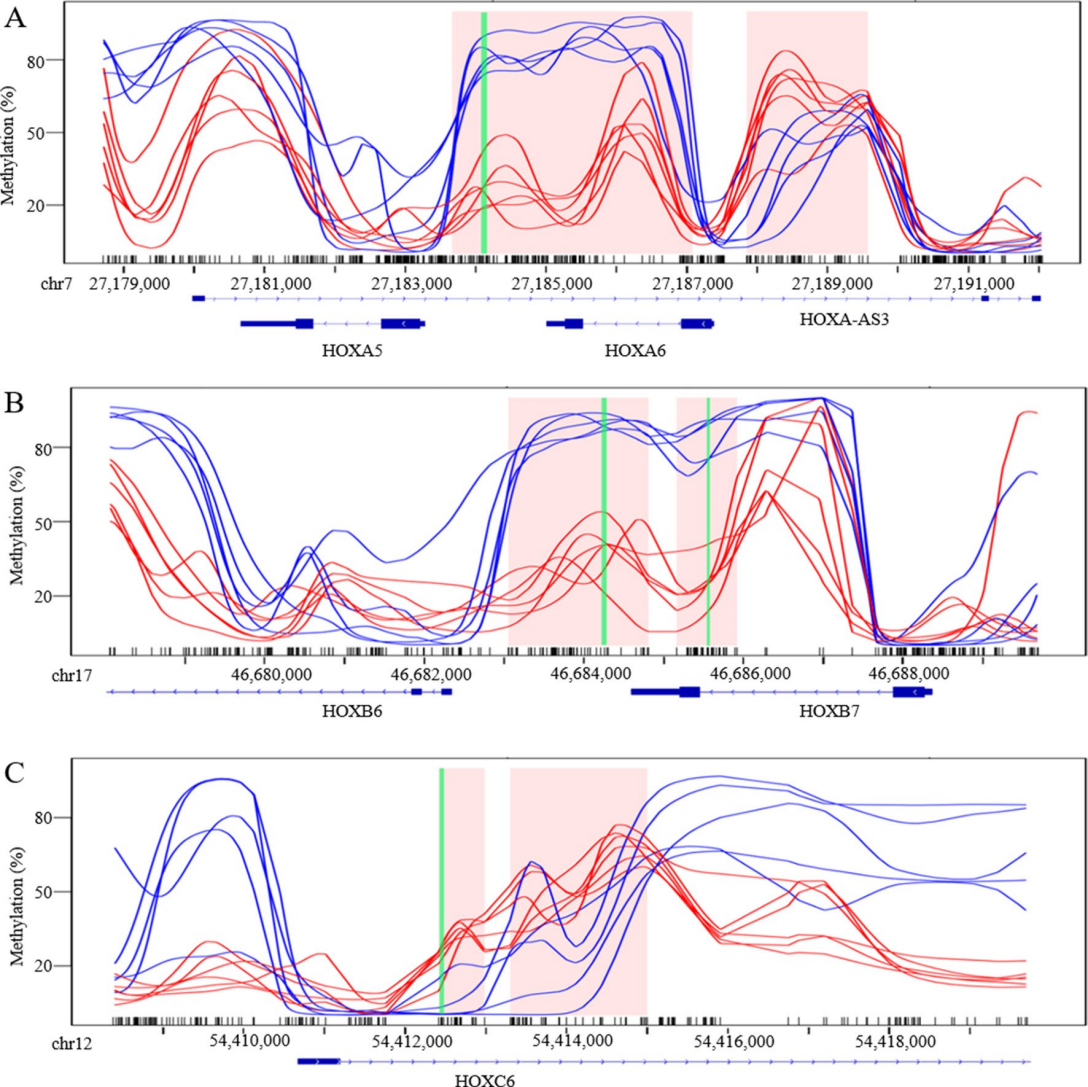
Representative DMRs identified by WGBS. Smoothed methylation values were plotted with identified DMRs indicated in pink. Regions replicated by bisulfite pyrosequencing (refer to Fig. 3) are highlighted in green. Plots of the** a** *HOXA* cluster, ** b** *HOXB* cluster, and ** c** *HOXC* cluster

**Fig. 3:**
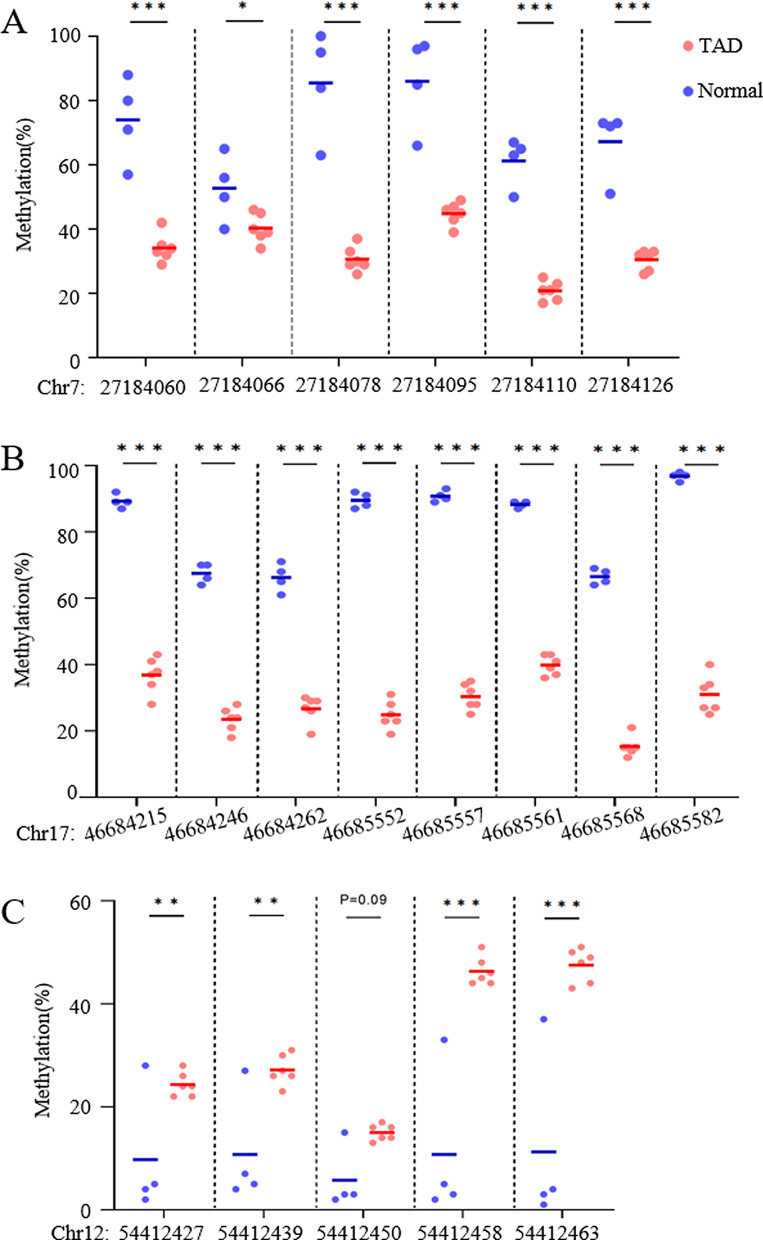
Replication of DMRs by bisulfite pyrosequencing from an independent sample set (n = 10). DMRs in the **a** *HOXA5* region, **b** *HOXB6* region, and **c** *HOXC6* region. (*P- value < 0.05, **P-value < 0.01, ***P-value < 0.001; Student’s t-test, unpaired and two-sided)

We noticed that Homeobox (*Hox*) gene cluster was frequently observed in both hyper- and hypomethylated DMRs. Fifteen out of the top 200 DMRs are located within homeobox (*Hox*) gene clusters, including the *HOXA* cluster on chromosome 7 (Fig. 2a), *HOXB* cluster on chromosome 17 (Fig. 2b), *HOXC* cluster on chromosome 12 (Fig. 2c) and *HOXD* cluster. *Hox* genes are critical in the regulation of cell proliferation, differentiation, and migration and have been implicated in cardiovascular development and disease [21, 22]. Thus, the potential roles of *Hox* genes in TAD were further evaluated. The observed DMR methylation differences were first replicated using bisulfite pyrosequencing, with 10 additional (6 TAD and 4 controls) ascending aortic tissue samples. Four DMRs were evaluated within three different *Hox* gene clusters, resulting in 19 CpGs being examined. Statistically significant methylation differences were observed between the TAD patients and healthy controls (Fig. 3), thus further confirming the WGBS findings.

### Reduced expression of *HOXA5*, *HOXB6* and *HOXC6* in TAD samples

**Fig. 4:**
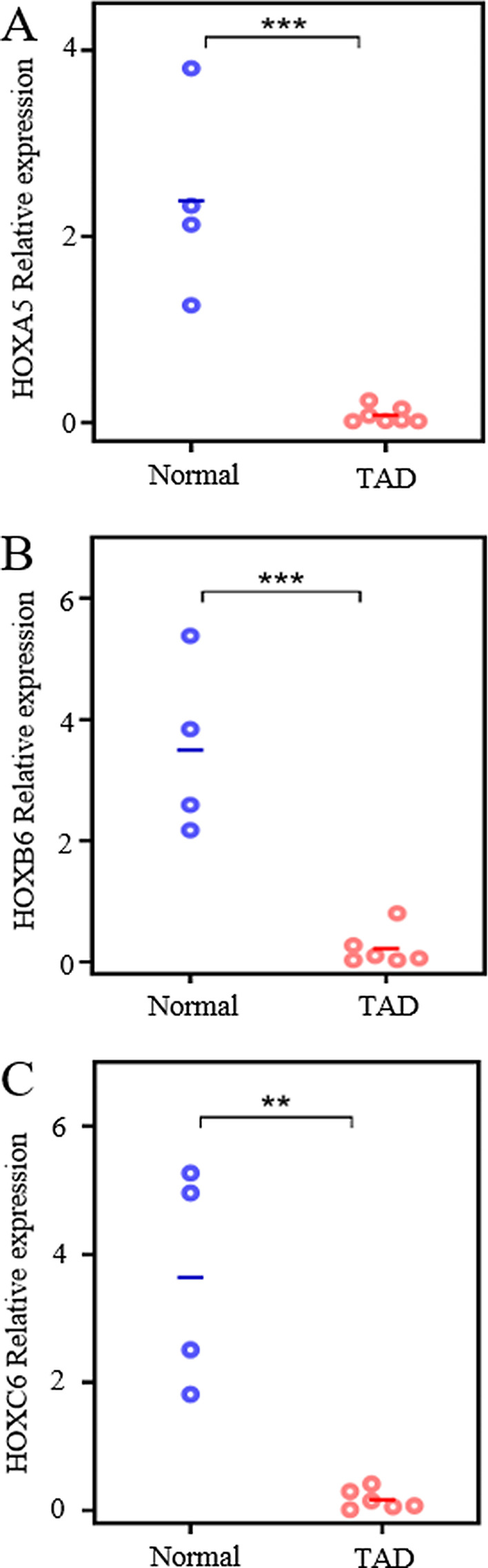
Differential expression of *Hox* family genes in TAD patients evaluated by qPCR. Genes examined include **a** *HOXA5*, (B) *HOXB6*, and (C) *HOXC6*. (*P- value < 0.05, ** *P*-value < 0.01, *** *P*-value < 0.001; Student’s *t*-test, unpaired and two-sided)

DNA methylation in different genomic region has diverse impact on gene expression. Two of the DMRs are located within the promoter regions of *HOXA5* and *HOXB6*, and one located at the intron of HOXC6. Thus, we evaluated whether the altered DNA methylation seen in TAD subsequently alters the expression of these genes. *HOXA5*, *HOXB6* and *HOXC6* expression levels are all significantly reduced in TAD patients when compared to the healthy controls (Fig. 4). Moreover, the expression levels of *HOXA5* and *HOXB6* are significantly correlated with their methylation levels, while the correlation for *HOXC6* is relatively low (Additional file 1: Figure S4A, B, C, D). DNA methylation in promoter region was generally known to be negatively correlated with gene repression; however, increasing evidence suggested that it may also contribute to gene activation [23] or splicing regulation [24]. The detail mechanism of how DMRs lead to Hox gene expression change, and whether it finally altered Hox transcription factors abundance in TAD need further investigation. These findings suggest that TAD associated DNA methylation alterations may reduce *Hox* family gene expression and may play a role in TAD pathogenesis.

### Cell-free DNA methylation as a potential biomarker for TAD

**Fig. 5:**
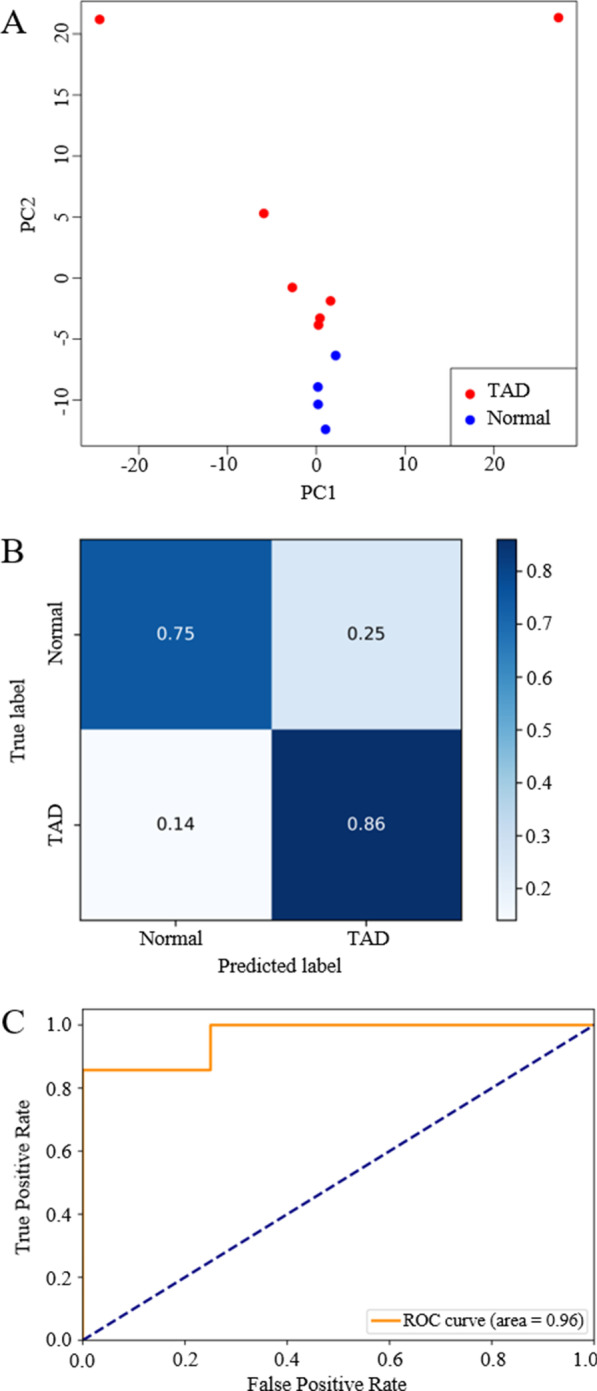
Methylation signatures from cfDNA as a biomarker for TAD diagnosis. **a** Principal component analysis of cfDNA methylation data on tDMRs (red dots indicate TAD patient and blue indicate healthy people, *P* value = 0.037 from PERMANOVA analysis). The top 50 DMRs with the largest cfDNA methylation variance contributing to the top two principal components were selected for model training. Optimized random-forest model was evaluated with LOOCV, and the confusion matrix (**b**) and the ROC curve (**c**) were plotted to assess the performance of the model (AUC = 0.96). Sensitivity: 86%, specificity: 75%. (LOOCV: leave-one-out cross validation, ROC: Receiver operating characteristic, AUC: area under curve)

Given distinct DNA methylation pattern between TADs and healthy controls (Fig. 1c), we continued to investigate whether the DNA methylation signature can be used as a biomarker to distinguish TAD from normal. Even though DNA methylation is tissue specific, numerous studies showed that methylation from plasma cfDNA, which is released from apoptotic cells, can be used as a surrogate measurement for primary tissues for disease prediction [12–14]. We thus performed WGBS for plasma cfDNA from another independent set of 7 TAD patients and 4 age-matched healthy controls (Additional file 1: Table S4, S5) and identified 45,462 DMRs. A total of 3982 DMRs (8.8%) are overlapped with DMRs identified from aortic tissues (tDMRs). A total of 2326 DMRs covering 12,856 differentially methylated sites (DMSs) showed the same methylation pattern as tDMRs (Additional file 3: Table S6). Among them, 34 DMRs were overlapped with top 300 tDMRs. This result indicates that plasma cfDNA may contain information released from aortic tissues. A principal component analysis (PCA) based on tDMRs showed a clear separation between the two groups (Fig. 5a, *P*-value = 0.037). The top 100 tDMRs with the largest methylation variance for cfDNA in PCA analysis were enriched in heart morphogenesis and cardiac development (Additional file 1: Figure S5), suggesting that, to a certain extent, the methylation signatures from cfDNA explained the condition of cardiovascular system and may be used for TAD prediction. To further evaluate the performance of cfDNA methylation on TAD prediction, we carried out a set of machine-learning analysis. Top 50 tDMRs with the largest methylation variance for cfDNA were first selected based on PCA (detail information are shown in Additional file 4: Table S7). Unsupervised clustering with methylation level from cfDNA on these 50 tDMRs successfully separated healthy ones from TADs (Additional file 1: Figure S6). Then, a random-forest-based prediction model was trained on methylation level from cfDNA on these 50 tDMRs using leave-one-out cross validation. The performance of the model was assessed by confusion matrix (86% sensitivity and 75% specificity; Fig. 5b) and F score (Additional file 1: Table S8). ROC curve shows that high specificity and sensitivity were achieved in patient classification (AUC = 0.96, Fig. 5c), indicating that a disease-specific signal was efficiently extracted from cfDNA methylation pattern and can be used for TAD detection. In summary, these results proved that methylation signal from cfDNA can be served as a potential biomarker for TAD detection.

## Discussion

In this study, a genome-wide DNA methylation analysis was performed to evaluate TAD associated epigenetic changes. While tissue samples were collected from the same collection site in both the TAD and control samples, numerous DMRs were identified and their associated genes enriched in the areas of vasculature and heart development. Among them, epigenetic changes in *Hox* gene clusters that altered *HOXA5*, *HOXB6* and *HOXC6* gene expression were noted. Similar to the methylation alteration in the primary tissues, methylation signatures from plasma cfDNA can be used in TAD patient classification. The findings presented herein suggest that, first, the epigenetic regulation of *Hox* genes in the aortic tissues may be associated with TAD pathogenesis, and second, cfDNA methylation can be used as a non-invasive biomarker for TAD detection.

CVDs are the leading cause of mortality worldwide [25], partially due to successful prevention and treatment being challenging. CVDs etiology is multifaceted, with known genetic and environmental risk factors only explaining a small aspect of onset and development. Thus, exploration into other factors, such as epigenetics, is necessary to overcome this obstacle. Epigenetics examines heritable changes that affect gene expression without altering the DNA sequence itself. When investigating epigenetic changes in association with genetic and environmental risk factors, a more complete insight into the pathophysiologic mechanisms underlying CVDs should be attainable [26].

DNA methylation is the most common and well-characterized epigenetic modification. However, studies focusing on the role of DNA methylation in CVDs have only been performed in atherosclerosis, hypertension, heart failure and calcific aortic valve disease. In atherosclerosis, a number of atherosclerosis-related genes are directly regulated by DNA methylation modifications [27]. In dilated cardiomyopathy, distinct methylation loci were identified in patients’ myocardial tissue and peripheral blood [28]. These methylation loci could potentially serve as epigenetic signatures for dilated cardiomyopathy and potential biomarkers for heart failure. For calcific aortic valve disease, a dysregulation of DNA methylation in the H19 promoter stimulates a more osteogenic phenotype by interfering with NOTCH1 expression [18].

In contrast to other CVDs, epigenetic studies examining aortic pathology are relatively sparse. While some studies have shown that DNA methylation is involved in inflammation, cellular proliferation and extracellular matrix (ECM) degradation during the development and progression of an abdominal aortic aneurysm [29–31], little is known regarding how epigenetic modifications may affect TAD pathogenesis, especially the devastating Stanford type A. Thus, we performed the first genome-wide study to investigate DNA methylation alterations in TAD using WGBS and observed an altered profile to be associated with TAD patients. This finding is consistent with a recent publication that investigated DNA methylation difference between TAD and bicuspid aortic valve with thoracic aortic dilatation [32]. However, that study utilized the Illumina 450 K beadchip platform, which only covers less than 3% of total CpG sites in the human genome. The present study provides an unbiased genome-wide profiling of DNA methylation at a single base resolution, and, by comparing to normal tissues, our work may provide additional insights into TAD pathogenesis.

Consistent with previous publications [33–36], localized SMC rarefaction, collagen deposition and elastin degradation were observed in TAD (Stanford type A) patients via histopathologic evaluation. Since SMCs produce many ECM proteins, such as elastin and collagen, additional investigation into whether epigenetic dis-regulation in SMCs may lead to pathological ECM alternations in TAD patients is needed.

Despite histopathologic evaluations showing no clear distinctions in primary cell type between the TAD and control samples, numerous DMRs were identified, with some enriched in *Hox* gene clusters, which are related to vasculature and heart development. Further analysis showed that *HOXA5*, *HOXB6* and *HOXC6* expression was significantly down-regulated in TAD patients.

*Hox* genes encode transcription factors that regulate the expression of lineage-specific genes and can control cell proliferation, differentiation and migration [37–42]. Several *Hox* genes are expressed in the cardiovascular system during embryogenesis and persist after birth [43]. Moreover, *Hox* gene expression has been detected in adult and fetal human aortic smooth muscle tissues [44]. In *Hoxa3*-null transgenic mice, multiple defects, including heart wall malformations, persistent patent ductus arteriosus, and stenosis of the aortic valve, contribute to death shortly after birth. Furthermore, all of the transgenic mice had thin, poorly developed aortic walls [38]. These findings suggest that *Hox* family genes may serve an important role in the development and maintenance of normal cardiovascular structures and functions.

In addition to potential roles in heart development and maintenance, *Hox* genes are also important regulators of SMC phenotypic plasticity. In C3H10T1/2 cells, a multipotent cell line able to differentiate into vascular SMCs and osteogenic and chondrogenic lineages, *HOXB7* overexpression resulted in increased proliferation, induction of a vascular SMC-like morphology, and the expression of early vascular SMC markers [45]. Klein and colleagues [46] also showed the importance of *HOXB7*, *HOXC6*, and *HOXC8* in the regulation of the differentiation of human vascular wall-resident multipotent stem cells (VW-MPSC) into SMCs via epigenetic mechanisms. These findings are consistent with the hypothesis that *Hox* family genes are potential regulators of SMC phenotypic alterations and thus associated with TAD pathogenesis.

In addition to the *Hox* family genes, GO analysis indicated that many other genes associated with vasculature and heart development are also affected in TAD patients. This finding is consistent with our previous array-based study that implicated a dedifferentiated SMC phenotype in aortic dissection patients [47]. Taken together, these findings suggest that the altered epigenetic regulation of *Hox* family genes potentially impacts aortic integrity in TAD due to the defects of SMC differentiation.

TAD is a severe disease with high mortality. Early diagnosis of TAD is imperative, especially under certain circumstance when imaging is unavailable. Moreover, TAD shared similar clinical symptom with segment elevation myocardial infarction and pulmonary embolism, which may lead to misdiagnosis. All of these indicate that an efficient and high accuracy biomarker was needed for TAD detection. Various protein markers were identified for TAD detection, such as D-Dimer, soluble ST2 and smooth muscle myosin heavy chain (smMHC) [8, 9]. However, most of them are still under study with limited clinical applications. In recent years, liquid biopsy was widely applied in tumor detection and non-invasive prenatal testing based on somatic mutation or chromosome variation. But low sensitivity and frequency make it quite challenging for general application. By contrast, epigenetic alterations are large-scaled and tissue-specific, therefore, have a greater potential in disease detection. However, to our knowledge, few works were reported to explore the application of epigenetic biomarkers in TAD detection. Here, we constructed a TAD prediction model based on the methylation profile of cfDNA. To reduce the noise from plasma, tDMRs were selected as prediction features. Compared with aortic tissue, Hox genes showed no significant difference between TADs and healthy controls in plasma (Additional file 1: Figure S7). This is not surprising considering cfDNA is a combination of DNAs from many different tissues. TAD is a complicated disease with various complications, which may lead towards severe damage to different tissues. These impaired tissues with various DNA methylation signatures on Hox genes will release their DNA into plasma and this may eventually mask the signals derived from aorta. Thus, we performed unsupervised analysis for cfDNA methylation data and took 50 tDMRs with largest variance for model training. Considering the limited sample size, we trained the model with 10 samples and left one for validation each time, and this process was repeated 11 times to make the best use of all samples. Finally, our model achieved 86% sensitivity and 75% specificity. The results were comparable with some verified biomarkers, such as D-dimer (sensitivity: 97%, specificity: 40–100%) [48, 49] which has been applied for TAD diagnosis in clinical. Compared with protein-based biomarkers, cfDNA methylation signature showed high tissue and disease specificity. As for transcriptional or mutation-based detection, only few miRNA were reported to have high accuracy in TAD prediction [50]; however, further optimization or verification was needed. Our work demonstrated a promising role for cfDNA methylation profile in TAD detection, as well as potentially in early diagnosis and disease discrimination. Meanwhile, a replication with much more samples will be needed before this can be used eventually for the clinical purpose.

One limitation of this study is the small sample size used in the WGBS analysis and prediction model training. Over the past years, few studies have been focused on clinical analysis of epigenetic regulation in type A TAD; especially for cfDNA methylation in TAD, there were no other dataset can be referred. One potential reason is the difficulty of sample collection, especially for healthy controls. Organ donation is the only way to obtain normal ascending aorta. Moreover, once onset, type A TAD progresses quickly, which needs immediate medical intervention. However, after intervention, either aorta tissues or bloods are on longer suitable for pathogenic analysis. In recent years, several WGBS studies on similar clinical scenarios with limited sample size (around 2–6 samples for each group) were performed and all of them achieved convincing results [51–53]. In this study, in order to address the limitation of small sample size, findings were replicated using an independent sample set with bisulfite pyrosequencing, and leave-one-out cross-validation was used in model training to fully take advantage of the limited number of samples. Nevertheless, small sample size is one clear limitation of this study, which greatly affects the power to identify DMRs with small effect size and impairs our ability to obtain a comprehensive picture of epigenetic changes in TAD. That being said, as the first genome-wide study to investigate DNA methylation alterations in TAD, this work provides valuable insights on the important role of DNA methylation in TAD pathogenesis.

Another limitation of this study is the possibility of contamination from other cell types cannot be excluded and may have affected the DNA methylation analysis. However, histopathologic evaluations showed that SMCs were the primary cell type in both TAD and control samples, and thus potential contamination from other cell types should be limited. Third, while this study focused on identifying differential DNA methylation associated with TAD, it is hard to know whether these altered epigenetic changes are causal factors of disease etiology, or simply consequences of TAD. To fully understand the molecular mechanism of these epigenetic changes that occur during TAD pathogenesis, additional functional experiments using cell cultures or even animal models are necessary. Nevertheless, these epigenetic changes may be potential biomarkers for TAD detection.

In conclusion, our findings revealed an altered DNA methylation pattern in TAD. Also, these results suggest that epigenetic regulation of *Hox* genes in the aortic tissues may be crucial in the maintenance of aortic integrity and be associated with TAD pathogenesis. Furthermore, methylation signatures from cfDNA can distinguish TAD from normal, demonstrating the potential utility of cfDNA methylation profile for non-invasive diagnosis in TAD.

## Methods

### Sample collection and ethics statement

Thoracic aortic fragments were dissected from nearby primary tear sites (ascending aortas above the sinuses of Valsalva) in 12 Stanford type A TAD patients who were undergoing surgical repair. All patients showed no signs of traumatic aortic injuries and were not previously diagnosed with Ehlers-Danlos syndrome, Marfan syndrome, or other connective tissue disorders. Additionally, ascending aortic tissues were obtained from 10 healthy multi-organ donors (controls) whose deaths were not associated with a vascular disease. The included patients and controls were closely matched in age and gender, with the demographic and clinical characteristics noted (Table 1). Samples were collected as previously described [54], and written informed consent was obtained from each participant.

**Table 1:** Sample characteristics

	Samples for WGBS* TAD^†^ (*n* = 6) Controls (*n* = 6)		Samples for replication TAD (*n* = 6) Controls (*n* = 4)	
Age, years	45.3 ± 11.6	42.8 ± 5.8^‡^	41.8 ± 8.0	36.5 ± 15.2^§^
Gender, male: female	5:1	6:0	5:1	3:1
Stanford classification				
Type A	6 (100%)	–	6 (100%)	–
Type B	0	–	0	–
Dissection staging				
Acute phase	6 (100%)	–	6 (100%)	–
Chronic phase	0	–	0	–
Arteritis	0	0	0	0
Bicuspid aortic valve	0	0	0	0
Family history of aortic diseases	0	0	0	0
Rupture	0	–	0	–
Maximal aortic diameter (mm)	5.2 ± 1.5	–	5.7 ± 3.3	–
Emergent operation	6 (100%)	–	6 (100%)	–
Elective operation	0	–	0	–

^*^WGBS whole genome bisulfite sequencing

^†^*TAD* thoracic aortic dissection

^‡^*P *value = 0.646

^§^*P* value = 0.497

For cfDNA analyses, blood samples were collected from another independent set of 7 type A TAD patients before any medical treatment and 4 healthy controls. Written informed consent was also obtained from each participant. The study was conducted following the principles outlined in the Declaration of Helsinki and approved by the Ethics Committee of Zhongshan Hospital, Fudan University, Shanghai, China (Approval No. B2018-040R).

### Histopathology

Thoracic aortic medial samples were isolated ex vivo, fixed in 10% neutral buffered formalin and embedded. The adventitia was gently removed to eliminate associated fatty tissues and endothelium. The tunica medial samples were divided into pieces for histologic examinations and biochemical analyses. Prior to staining, samples were deparaffinized, rehydrated and then stained with hematoxylin and eosin. Masson’s and Gomori's trichrome stains were also performed to evaluate aortic histologic structures.

### Whole-genome bisulfite sequencing (WGBS)

DNA of thoracic aortic tissues was obtained from TADs (*n* = 6) and normal controls (*n* = 6). DNA was extracted using a DNeasy Blood and Tissue Kit (Qiagen, Valencia, CA, USA) according to the manufacturer’s protocols. Each sample was then spiked with 1% unmethylated lambda DNA (Promega, Madison, WI, USA) to evaluate the bisulfite conversion efficiency. The genomic DNA (500 ng) was then fragmented to an average size of 250 bp using a Covaris M220 ultrasonicator (Covaris, Woburn, MA, USA). End repair and methylated adaptor ligation was performed with NEBNext Ultra End Repair/dA-Tailing Module, Ligation Module and NEBNext Multiplex Oligos for Illumina (Methylated Adaptor, Index Primers Set 1; New England Biolabs, Ipswich, MA, USA). DNA fragments between 300 and 400 bp were selected for library construction using Ampure XP beads (Beckman Coulter, Brea, CA, USA). Samples then underwent bisulfite conversion using an EZ DNA Methylation kit (Zymo Research, Irvine, CA, USA), with modified single stranded DNA fragments amplified using a Kapa HiFi U + HotStart ReadyMix (Kapa Biosystems, Wilmington, MA, USA) with primers (NEBNext Multiplex Oligos for Illumina). A final size selection was performed to enrich the library to a range between 300 and 500 bp. Constructed libraries were evaluated on an Agilent 2100 Bioanalyzer (Agilent Technologies, Santa Clara, CA, USA) to assess quality and sequenced on an Illumina HiSeq X Ten or NovaSeq (Illumina, San Diego, CA, USA) using the 150-bp paired-end mode.

Cell-free DNA was obtained from 7 TADs and 4 normal controls. Whole blood was collected in EDTA tubes and processed within 4 h after the blood drawn. 1–3 ml plasma was separated with centrifuge twice at 1600 g for 10 min, and then 16,000 g for 10 min at 4 °C. DNA was isolated from plasma using the Qiagen Circulating Nucleic Acids Kit (Qiagen, Valencia, CA, USA) according to the manufacturer’s instructions. The quality of cfDNA was assessed by Agilent 2100 Bioanalyzer. The WGBS libraries from cfDNA were prepared with similar procedures as the tissue DNA with minor modifications: (1) 10 ng cfDNA with no fragmentation was used for library preparation, and (2) after final amplification, DNA libraries were only purified with XP beads to remove primer dimers.

### WGBS data analysis

Sequencing reads were analyzed using Bismark v.0.22.1 [55]. Briefly, reads were aligned to the human genome (hg19), together with the lambda phage genome, using Bowtie2 v.2.2.3. Following alignment, reads from PCR duplication were removed, methylation measurements for each CpG site were obtained, and the bisulfite conversion rates were calculated based on the spiked-in unmethylated lambda phage DNA. For tissue samples, one control sample with bisulfite conversion rate lower than 90% (sample: N2) was removed.

To identify DMRs from aortic tissue samples, the bsseq package in BSmooth was employed [56]. Only CpGs appearing at least twice in at least three samples per group were included in the analysis. DMRs were identified using a smooth window containing either 70 CpGs or a width of 1 kb, whichever was larger. Next, regions satisfying the following criteria were deemed putative DMRs: (1) a t-statistic with a qcutoff between a range of (0.025,0.975); (2) must contain at least 3 CpG sites; (3) must have a methylation difference of at least 10%; and (4) must located on autosomes.

### The pathway enrichment analysis

The tissue DMRs were annotated to their nearest genes and then hypergeometric test was performed with a focus on biological processes using the Molecular Signatures Database and the R package clusterProfiler v.3.6.0 enricher function with default parameters (*P*-value cut-off = 0.05; OrgDb = org.Hs.eg.db; and p.adjust method = “BH”). Pathway enrichment analysis was performed with Kyoto Encyclopedia of Genes and Genomes (KEGG) database (*P*-value cut-off = 0.05) and R package clusterProfiler v.3.6.0.

### DNA and RNA extraction

DNA from an additional 10 independent thoracic aortic tissue samples (6 TAD patients and 4 healthy controls) was used for downstream replication. The thoracic aortic tissues were further cut into small pieces for either DNA or RNA extraction. DNA was extracted using phenol–chloroform and purified with ethanol precipitation. Total RNA was extracted using TRIzol reagent (ThermoFisher, Waltham, MA, USA) according to the manufacturer’s protocol.

### Bisulfite pyrosequencing

Genomic DNA (500 ng) was treated with sodium bisulfite using an EZ DNA Methylation kit (Zymo Research, Irvine, CA, USA), with samples eluted in 20 ul of elution buffer according to the manufacturer’s instructions. Converted genomic DNA was then diluted in water (1:5), with 2.5 ul then used for nested PCR amplification with Premix Taq HotStart (Takara). PCR primers (Additional file 1: Table S1) were designed using the PyroMark Assay Design software (Qiagen, Valencia, CA, USA), and amplicons were confirmed via agarose gel. Pyrosequencing was performed using the PyroMark Q24 System (Qiagen, Valencia, CA, USA), and results were automatically analyzed by the PyroMark Q24 software.

### Quantitative real-time reverse transcription polymerase chain reaction (qPCR)

Total RNA (1 ug) was reverse transcribed into cDNA using a PrimeScript RT reagent kit (Takara). Quantitative real-time PCR was performed with 2 × SYBR Green qPCR Master Mix (Bimake) on a LightCycler 480 system (Roche). All samples were analyzed with three technical replicates, and glyceraldehyde phosphate dehydrogenase (GAPDH) levels were used as an internal control. (Primer sequences are listed in (Additional file 1: Table S1).

### Prediction model for methylation from cfDNA

Methylation level for cfDNA was calculated as the sum of methylated reads divided by the sum of total reads for each CpG site. CfDNA methylation level located in tissue-derived DMRs (tDMRs) was extracted for downstream analysis. Prediction model was built in three steps, and all steps were conducted using python package scikit-learn [57]. First, feature selection was performed using principal component analysis (PCA) to identify the top 50 tDMRs with the largest cfDNA methylation variance contributing to the top two principal components. Second, prediction model was trained and evaluated based on random-forest algorithm and leave-one-out-cross-validation (LOOCV) method. Specifically, eleven cfDNA samples were separated into ten samples as the test set and the other one left as the validation set. Random-forest model was optimized through five times cross-validation [57] using the test set. After that, model performance was evaluated using the prediction result on the validation set. This process was repeated eleven times, with different sample left in the validation set each time. Third, all the prediction results were summarized to generate the confusion matrix to evaluate the true positive rate, true negative rate, false positive rate and false negative rate. Based on that, receiver operator characteristic (ROC) curve was plotted.

#### Statistical analysis

Bisulfite pyrosequencing and qPCR results were compared using an unpaired two-tailed Student’s *t* test, with a *P* value < 0.05 considered statistically significant.

## Supplementary Information

**Additional file 1**. Supplementary materials (Figures S1-S7; Table S1, Table S2, Table S4, Table S5 and Table S8)

**Additional file 2**. **Table S3.** Identified differentially methylated regions in tissue.

**Additional file 3**. **Table S6.** Overlapped DMRs between cfDNA and tissue.

**Additional file 4**. **Table S7.** Tissue-derived differentially methylated regions (tDMRs) used for cfDNA classification.

## Data Availability

All data generated or analyzed in this study are included in this manuscript and its supplementary information files. Sequencing data and codes are available from the corresponding authors on reasonable request.

## Abbreviations

TAD: Thoracic aortic dissection
CVDs: Cardiovascular diseases
WGBS: Whole-genome bisulfite sequencing
DMRs: Differential methylated regions
Hox: Homebox
cfDNA: Cell-free DNA
ROC: Receiver operating characteristic
PCA: Principle component analysis
