# Supplementary material for: Altered DNA methylation pattern reveals epigenetic regulation of Hox genes in thoracic aortic dissection and serves as a biomarker in disease diagnosis
Source: Clin Epigenetics. 2021 Jun 8;13:124. doi: 10.1186/s13148-021-01110-9 (PMC8186232; doi:10.1186/s13148-021-01110-9)
Supplement: Supplementary file 1 — Additional file 1. Supplementary materials (Figures S1-S7; Table S1, Table S2, Table S4, Table S5 and Table S8) [file 13148_2021_1110_MOESM1_ESM.docx]

SUPPLEMENTARY MATERIALS


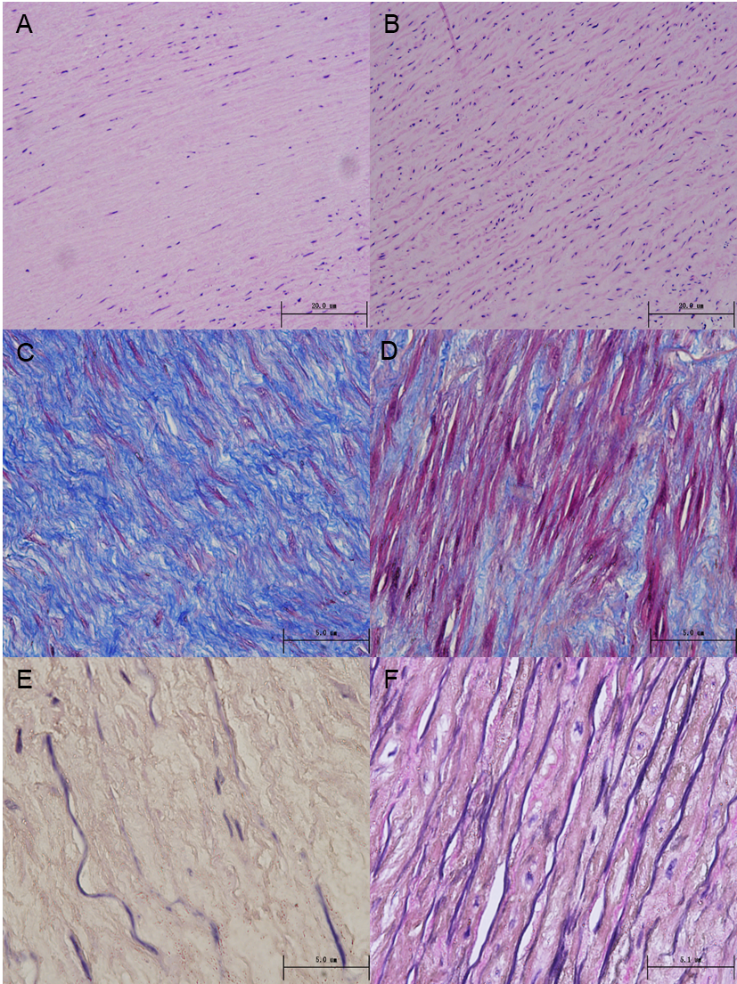


**Supplemental Figure 1.** **Histochemistry staining of thoracic aortic medial samples.**

H&E staining show localized smooth muscle cell rarefaction in the dissected media for TADs (A) compared to the controls (B). Masson’s trichrome staining show the elevated deposition of total collagens in the dissected media (C), collagens were stained blue and smooth muscle cells were stained red) compared to the controls (D). Gomori’s staining displayed the severe fragmentation and decrease of elastic fibers in the pathologic media (E, elastic fibers were stained violet or dark blue) compared to the controls (F). The scale bars in the lower right corner equals to 20µm (Objective magnification ×10) and 5µm (Objective magnification ×40).


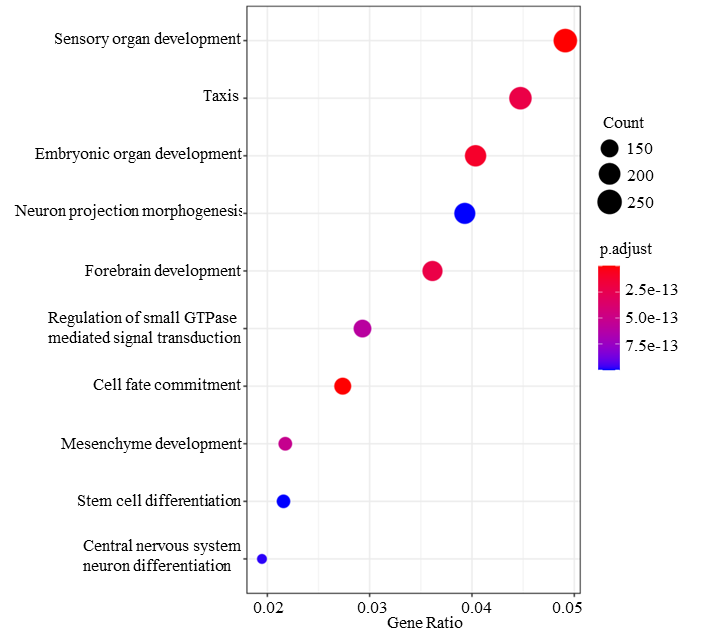


**Supplemental Figure 2**. **The gene ontology analysis with a focus on biological processes for the hypermethylated DMRs.**


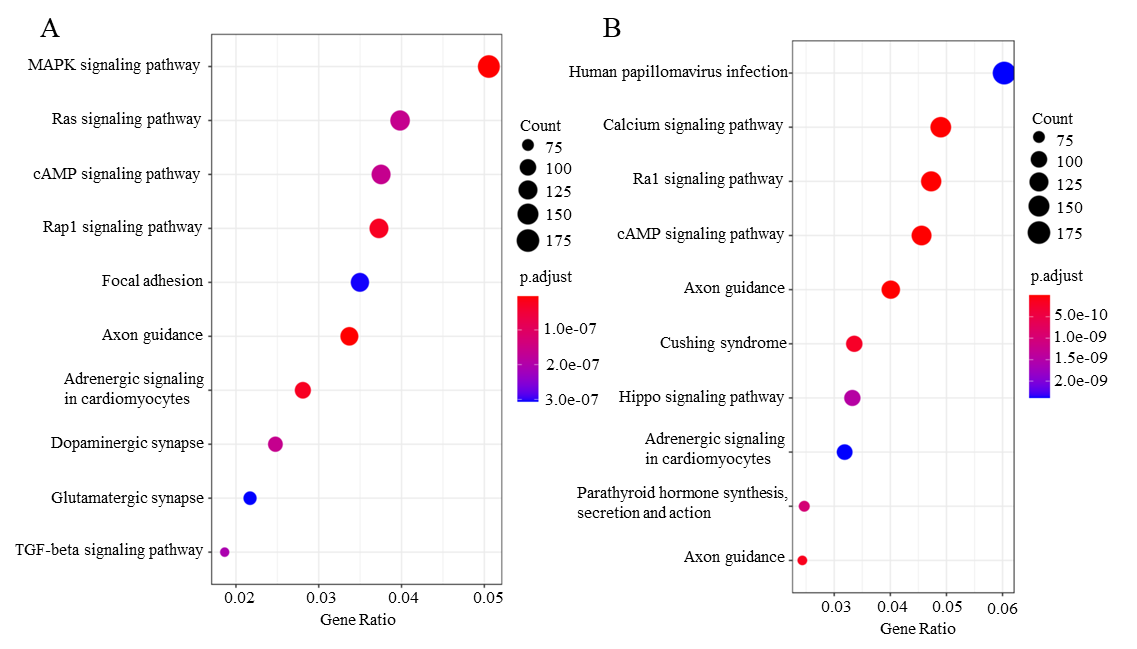


**Supplemental Figure 3. The pathway enrichment analysis.** The pathway enrichment analysis for the hypomethylated DMRs (A) and hypermethylated DMRs (B).


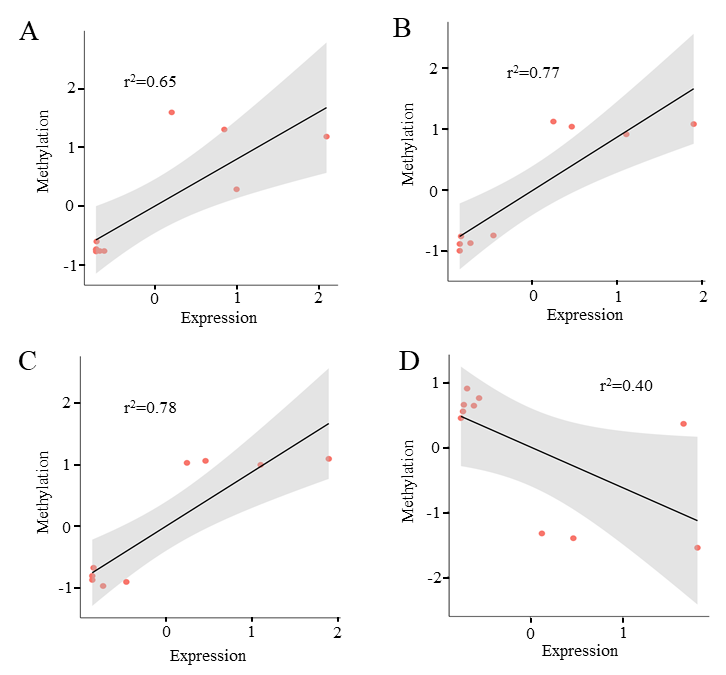


**Supplemental Figure 4. Correlation between expression and methylation in Hox.** The correlation-ship between expression and methylation in (A) HOXA5 (p-value=0.00506), (B,C) HOXB6 (p-value=0.00178, p-value=0.00173) and (D) HOXC6 (p-value=0.05152).


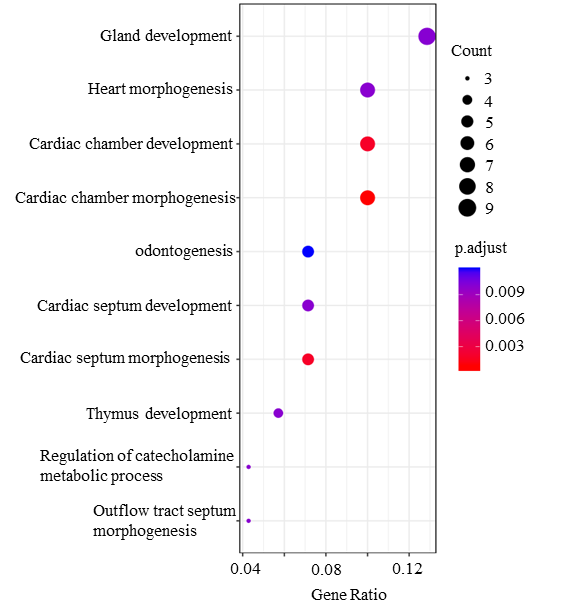


**Supplemental Figure 5.** The Gene ontology analysis for the top 100 tDMRs with the largest methylation variance for cfDNA in PCA analysis.


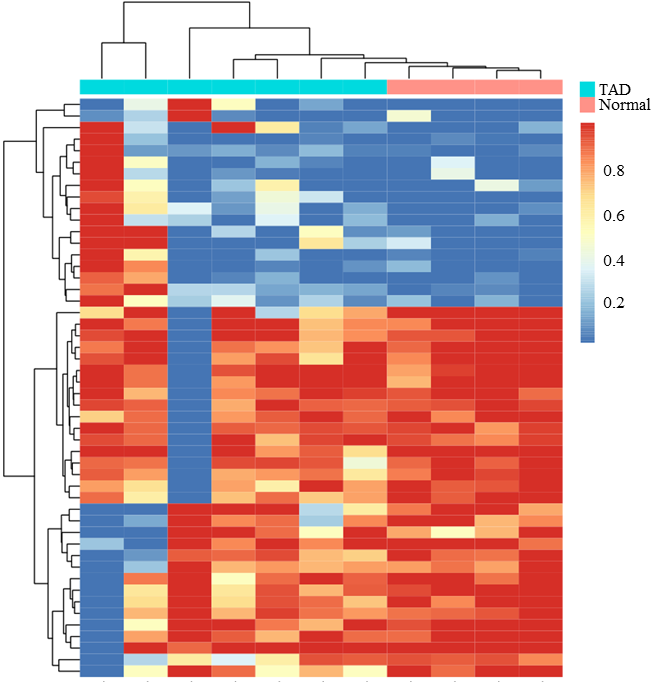


**Supplemental Figure 6**. **Unsupervised clustering of cfDNA samples.** Unsupervised clustering using top 50 tDMRs identified from PCA. The top bar indicated patient (blue) and healthy control (red). The level of color indicated methylation level. (tDMRs: tissue-derived DMRs)


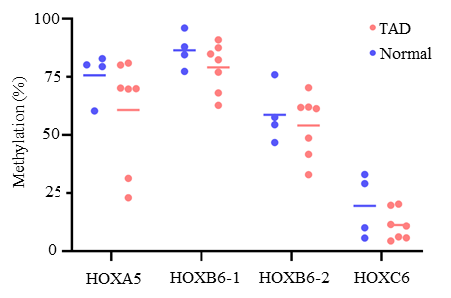


**Supplemental Figure 7. Average methylation level in Hox gene related DMRs (Figure 2. highlight with green) in plasma sample.**

| **Bisulfite pyrosequencing** | | | |
| --- | --- | --- | --- |
| **Regions (hg19)** | **Gene** | **Primer** | **Sequence (5' → 3')** |
| chr7: 27184057-27184132 | *HOXA5* | Forward | GTTAATTGGAGAAATAAATTTTGTT |
|  |  | Reverse | AACTACAAAAAACCTAAACCCACTC |
|  |  | Nested forward | GTATGAGGGATAGTGTTTTTG |
|  |  | Nested reverse | /5Biosg/ACCCTATAATAAAAATTTTCCAAACT |
|  |  | Sequencing(F) | AAATTTTATATATTTTTATA |
| chr17:46684212-46684285 | *HOXB6* | Forward | TGTGTTTGGGTTGTAGTTTAGA |
|  |  | Reverse | ATCACAAAAAAACACTCATACCCATATA |
|  |  | Nested forward | GGGTTTGTGAAATTGTGAGGTT |
|  |  | Nested reverse | /5Biosg/CACAAAAAAACACTCATACCCATAT |
|  |  | Sequencing(F) | TGAATGTGTGTTTATTTGTG |
| chr17:46685549-46685671 | *HOXB6* | Forward | AGTATTTAGAGGAAAATTAGTGAGTAAAG |
|  |  | Reverse | TTCTTCTACCTCAACCTCCTAAATAATT |
|  |  | Nested forward | GGTGGGTTTAAATTTGTAATTTTAGTA |
|  |  | Nested reverse | /5Biosg/CTTCTACCTCAACCTCCTAAATAA |
|  |  | Sequencing(F) | TAATTTTAGTATTTTGGGAG |
| chr12:54412403-54412442 | *HOXC6* | Forward | ATGGTTTTATAATATAGTGGTTTAATTT |
|  |  | Reverse | AAATAAATCTAACCCCCAAATTCT |
|  |  | Nested forward | GGTTTTGAATGTGTTGTAGTT |
|  |  | Nested reverse | /5Biosg/AAATCTAACCCCCAAATTCTCT |
|  |  | Sequencing(R) | TGTTGTAGTTTGATATATGATT |
| **RT-qPCR** | | | |
| **Gene** | **Primer** | **Sequence (5' → 3')** | |
| *HOXA5* | Forward | AGATCTACCCCTGGATGCGC | |
|  | Reverse | CCTTCTCCAGCTCCAGGGTC | |
| *HOXB6* | Forward | CTCCGGTCTACCCGTGGATG | |
|  | Reverse | CCGCGTCAGGTAGCGATTGT | |
| *HOXC6* | Forward | CCCCAGGACCAGAAAGCCAGTAT | |
|  | Reverse | ATTCCTTCTCCAGTTCCAGGGTCT | |
| *GAPDH* | Forward | TGCACCACCAACTGCTTAGC | |
|  | Reverse | GGCATGGACTGTGGTCATGAG | |

**Supplemental Table 1. Primer sequences used for bisulfite pyrosequencing and RT-PCR.**

| **Sample** | **Total Number of CpGs** | **Number of CpGs Covered** | **CpG Coverage**  **Rate** | **Depth** | **Average Methylation**  **Level** | **Bisulfite conversion**  **Rate** |
| --- | --- | --- | --- | --- | --- | --- |
| Nor1 | 56434896 | 44430183 | 0.787 | 10.17 | 0.772 | 0.971 |
| Nor2 | 56434896 | 39663113 | 0.703 | 8.50 | 0.778 | 0.968 |
| Nor5 | 56434896 | 43647886 | 0.773 | 10.92 | 0.779 | 0.972 |
| N2 | 56434896 | 32747358 | 0.580 | 6.96 | 0.781 | 0.884 |
| N4 | 56434896 | 47822422 | 0.847 | 11.59 | 0.755 | 0.995 |
| N6 | 56434896 | 49384992 | 0.875 | 10.18 | 0.742 | 0.997 |
| A-1 | 56434896 | 46462725 | 0.823 | 9.14 | 0.754 | 0.992 |
| A-2 | 56434896 | 40108064 | 0.711 | 6.27 | 0.752 | 0.995 |
| A-3 | 56434896 | 41860996 | 0.742 | 6.66 | 0.736 | 0.994 |
| T-719 | 56434896 | 44280570 | 0.785 | 10.44 | 0.791 | 0.979 |
| T-817 | 56434896 | 46665610 | 0.827 | 11.57 | 0.782 | 0.983 |
| T-S | 56434896 | 47244365 | 0.837 | 12.06 | 0.789 | 0.974 |

**Supplemental Table 2. Summary statistics for tissue WGBS.**

|  | **Samples for machine learning** | |
| --- | --- | --- |
|  | TAD (n = 7) | Controls (n = 4) |
| Age, y | 51±11.5 | 49.3±4.6* |
| Gender, male: female | 6:1 | 3:1 |
| Stanford Classification |  |  |
| type A | 7 (100%) | — |
| type B | 0 | — |
| Dissection staging |  |  |
| Acute phase | 7 (100%) | — |
| Chronic phase | 0 | — |
| Arteritis | 0 | 0 |
| Bicuspid aortic valve | 0 | 0 |
| Family history of aortic disease | 1 | 0 |
| Rupture | 0 | — |
| Maximal aortic diameter (mm) | 48±4.8 | — |
| Emergent operation | 7 (100%) | — |
| Elective operation | 0 | — |

**Supplemental Table 4. Blood sample characteristics.** *p-value = 0.782, student’s t-test.

| **Sample** | **Total Number of CpGs** | **Number of CpGs Covered** | **CpG Coverage Rate** | **Depth** | **Average Methylation Level** | **Bisulfite conversion Rate** |
| --- | --- | --- | --- | --- | --- | --- |
| test0 | 56434896 | 28186767 | 0.500 | 6.67 | 0.773 | 0.996 |
| test8 | 56434896 | 30299647 | 0.537 | 7.68 | 0.793 | 0.995 |
| test9 | 56434896 | 35460897 | 0.628 | 9.01 | 0.782 | 0.994 |
| test12 | 56434896 | 25090419 | 0.445 | 4.54 | 0.805 | 0.994 |
| test16 | 56434896 | 34324907 | 0.608 | 7.43 | 0.792 | 0.995 |
| test17 | 56434896 | 30590792 | 0.542 | 6.14 | 0.803 | 0.994 |
| test18 | 56434896 | 34699502 | 0.615 | 7.17 | 0.810 | 0.994 |
| test1 | 56434896 | 28388718 | 0.503 | 5.45 | 0.806 | 0.982 |
| test6 | 56434896 | 25560226 | 0.453 | 4.99 | 0.804 | 0.990 |
| test7 | 56434896 | 31007990 | 0.549 | 6.47 | 0.829 | 0.971 |
| test15 | 56434896 | 28254135 | 0.501 | 4.22 | 0.793 | 0.994 |

**Supplemental Table 5. Summary statistics for cfDNA WGBS.**

|  | Precision | Recall | F1-score | Support |
| --- | --- | --- | --- | --- |
| AD | 0.86 | 0.86 | 0.86 | 7 |
| Control | 0.75 | 0.75 | 0.75 | 4 |
| Accuracy | - | - | 0.82 | 11 |
| Macro avg | 0.80 | 0.80 | 0.80 | 11 |
| Weighted avg | 0.82 | 0.82 | 0.82 | 11 |

**Supplemental Table 8. Statistic value of prediction model.**
